# Current Burden of and Geographic Disparities in Liver Mortality and Access to Liver Transplant

**DOI:** 10.1001/jamanetworkopen.2024.39846

**Published:** 2024-10-18

**Authors:** Nicolas S. Rinella, William Charlton, Gautham Reddy, Paige McLean Diaz, Michael R. Charlton

**Affiliations:** 1 Division of Gastroenterology and Hepatology, University of Chicago, Chicago, Illinois; 2 Division of Orthopedics, Department of Surgery, University of Kentucky, Lexington

## Abstract

**Importance:**

Liver disease is a leading cause of mortality in the US. Liver transplant can be a lifesaving procedure for patients with severe liver disease.

**Objective:**

To assess temporal trends and geographic variance in liver-related mortality (LRM) and liver transplant in the US.

**Design, Setting, and Participants:**

In this cohort study, the frequencies and geographic variance of LRM in 2018 and 2021 were obtained in a cross-sectional analysis of the Underlying Cause of Death data available on the Centers for Disease Control and Prevention’s Epidemiologic Research database. The number of livers donated and transplanted according to the state of residence of each donor and recipient were obtained from the United Network for Organ Sharing.

**Main Outcomes and Measures:**

Liver-related mortality in 2018 and 2021, overall and by state, as well as the liver transplant rate according to state of residence of recipient and donor.

**Results:**

Overall LRM in the US was 93 418 in 2021, with a crude rate of 28.1 per 100 000 individuals, an increase of 19.1% compared with rates seen immediately prior to the COVID-19 pandemic in 2018 (77 282 [23.6 per 100 000 individuals]). Liver-related mortality in 2021 varied several-fold between states, from 18.4 per 100 000 individuals per year in Utah to 65.9 per 100 000 individuals per year in New Mexico. The mean number of liver-related deaths per transplant from all donor sources (in state and out of state) was 7.2 in the lowest LRM quintile compared with 21.5 in the highest (95% CI, 12.1-16.6; SE, 1.1; *P* < .001). Ten states had no liver transplant center. Paradoxically, residents of states with the highest LRM had a much lower rate of liver transplant (at any location) from organs procured from in-state residents than states with the lowest LRM quintile (13.0% vs 35.2% in-state donors; 95% CI, 14.1%-30.3%; SE, 3.9%; *P* < .001).

**Conclusions and Relevance:**

This study suggests that rates of LRM have increased dramatically since the COVID-19 pandemic and vary several-fold between states. Rates of liver transplant are paradoxically lowest among residents living in states with the highest LRM. These findings highlight apparent geographic disparities in access to liver transplant that allocation policy cannot address.

## Key Points

**Question:**

Does access to liver transplant reflect variation in the frequency of liver-related mortality (LRM) in the US?

**Findings:**

This cohort study found that LRM has increased by 19.1% in the last 4 years, with several-fold variation between states; access to liver transplant varied inversely with LRM, with states with the highest LRM having lower transplant rates than states with the lowest LRM. Ten states, almost all relatively rural, had no liver transplant center.

**Meaning:**

These findings suggest a broad increase and substantial variance between states in LRM.

## Teaser

This cohort study assesses temporal trends and geographic variance in liver-related mortality and access to liver transplant in the US.

## Introduction

The fair distribution of solid organs continues to be a significant challenge for policymakers seeking to prioritize transplants for the sickest patients first while also fulfilling the goals of equitable access, which include minimizing geographic restrictions and reducing deaths from organ failure. Although more than 10% of the US population lives in rural areas, most transplant centers are in urban centers. This unequal distribution of transplant centers is associated with an increased risk of overall mortality.^1,2,3^ Recent modifications of organ allocation policy for donated livers have sought to reduce geographic disparities in access to transplants and, thereby, to reduce mortality from liver disease among patients listed for liver transplant.^4^ Acuity circles, for example, are the current iteration of allocation policy development in liver transplant. As for preceding iterations of allocation policy, acuity circles were intended to reduce geographic variance in Model for End-Stage Liver Disease (MELD) scores at transplant by replacing the donation service area model and their regional boundaries with concentric circles centering on the location of the donor hospital. The acuity circles policy added needed flexibility to the allocation process by removing these largely arbitrary regional boundaries from consideration. Early outcomes data demonstrate that since implementation of acuity circles, the proportion of patients receiving an organ transplant with a MELD score higher than 29 has increased.^5,6,7,8^ Although refinements of organ allocation policies have succeeded in reducing geographic variance in access to liver transplant, organ allocation policies do not address access to transplant centers. Thus, while waiting list mortality rates have generally decreased over time, the broader association with liver-related morality (LRM) is unclear.^7,9^ In addition, an increasing number of donor organs originate from states with the lowest transplant rates and highest LRM and that often have no available in-state transplant centers.^10^ Although sequential refinements of organ allocation policy have enhanced some measures of equity, including waiting list mortality and transplant rates among patients with high MELD scores on the waiting list, these metrics do not capture LRM, regional variation in LRM, or regional access to liver transplant. Ideally, a liver transplant would be equally available to all patients who might benefit from it, with the distribution of transplant centers and liver transplant procedures broadly reflecting the burden of LRM. Outcomes data associating liver transplant rates and donor organ use with geographic variance in LRM, however, remain sparse. To that end, this study evaluates apparent geographic equity by analyzing the frequencies of organ transplant by state with respect to LRM as provided by the Scientific Registry of Transplant Recipients (SRTR) and Centers for Disease Control and Prevention (CDC) Wide-Ranging Online Data for Epidemiologic Research (WONDER) databases. The state residence data of both organ donors and recipients were analyzed to uncover trends in LRM.

## Methods

Waiver of review of the study was granted by the institutional review board of the University of Chicago. The institutional review board of the University of Chicago recognizes that the analysis of deidentified, publicly available data, such as the CDC WONDER and UNOS datasets used for this analysis, does not constitute human participants research as defined at 45 CFR 46.102 and that it does not require institutional review board review. This cohort study adheres to the Strengthening the Reporting of Observational Studies in Epidemiology (STROBE) reporting guideline.

Mortality rates were obtained from the CDC WONDER database, which reports national mortality and population data produced by the National Center for Health Statistics CDC. Mortality information is based on a single underlying cause of death for all ages reported on death certificates from US citizens, collected by state registries, and provided to the National Vital Statistics System. Deaths and death rates are reported by 4-digit *International Statistical Classification of Diseases and Related Health Problems, Tenth Revision* (*ICD-10*) codes. Liver-related mortality was captured by *ICD-10* codes for all acute and chronic LRM: B15.0 (hepatitis A with hepatic coma); B16.0 (acute hepatitis B with delta-agent [coinfection] with hepatic coma); B16.2 (acute hepatitis B without delta-agent with hepatic coma); B17.1 (acute hepatitis C); B19.0 (unspecified viral hepatitis hepatic with coma); C22.0 (liver cell carcinoma–malignant neoplasms); C22.7 (other specified carcinomas of liver–malignant neoplasms); C22.9 (liver, unspecified–malignant neoplasms); I85.0 (esophageal varices with bleeding); I85.9 (esophageal varices without bleeding); I86.4 (gastric varices); K65.0 (acute peritonitis); K70-K76 (diseases of liver); and R18 (ascites). Caveats include the following:

1. The population figures for years 2022 and later are single-race and ethnicity estimates of the July 1 resident population, from the vintage 2022 postcensal series released by the US Census Bureau on June 22, 2023. The 2022 series is based on the Modified Blended Base produced by the US Census Bureau in lieu of the April 1, 2020, decennial population count. The Modified Blended Base consists of the blend of vintage 2020 postcensal population estimates for April 1, 2020; 2020 Demographic Analysis Estimates; and 2020 US Census data from the internal Census Edited File. The population figures for year 2021 are single-race estimates of the July 1 resident population, based on the Blended Base produced by the US Census Bureau in lieu of the April 1, 2020, decennial population count, from the vintage 2021 postcensal series released by the US Census Bureau on June 30, 2022. The population figures for year 2020 are single-race estimates of the July 1 resident population, from the vintage 2020 postcensal series based on the April 2010 Census, released by the US Census Bureau on July 27, 2021.^11^ The population figures for year 2019 are single-race estimates of the July 1 resident population, from the vintage 2019 postcensal series based on the April 2010 Census, released by the US Census Bureau on June 25, 2020. The population figures for year 2018 are single-race estimates of the July 1 resident population, from the vintage 2018 postcensal series based on the April 2010 Census, released by the US Census Bureau on June 20, 2019.^12^
2. The population figures used in the calculation of death rates for the age group “under 1 year” are the estimates of the resident population that is younger than 1 year of age.^12^
3. Connecticut population estimates for 2022 are reported for 9 planning regions as county equivalent areas, instead of the former 8 legacy counties in the vintage 2022 postcensal series released by the US Census Bureau on June 22, 2023. Populations estimates for the former counties are not available for 2022.^12^
4. Changes to cause of death classification affect reporting trends.^12^

For more information, refer to National Vital Statistics System–Mortality Data.^10,12^

### Statistical Analysis

The frequencies of livers exported as a proportion of all-source donor use (donor organ transplant for a state recipient from in-state and out-of-state sources) were obtained from the SRTR database, which collects transplant-related data from all transplant centers nationally.^12^ States were divided into quintiles by either LRM or frequencies of imported or exported livers for analysis. The highest and lowest quintiles of LRM were analyzed for in-state donor use (use of liver donated from an in-state resident to a recipient from the same state, even if transplant occurred in different states). For example, a patient residing in New Mexico who was evaluated, placed on the waiting list, and underwent liver transplant in Colorado would be reflected as a waiting list addition for New Mexico. A donor organ from a donor from any state other than New Mexico that was transplanted into a resident of New Mexico at a transplant center in any state would be reflected as out-of-state donor to in-state recipient (an imported liver). In-state donor use from all sources was also compared. Livers lacking a state origin or from international sources were omitted from consideration in the tabulation of liver frequencies. Two-tailed *t* tests, with the significance level set at *P* < .05, by MedCalc, version 23.0.2 (MedCalc Software Ltd) and R, version 4.41 (R Project for Statistical Computing) were used for the statistical analysis. Quintile-quintile plots were used to approximate normality, with LRM, deaths per all-source donor use, in-state donor use, percentage below the federal poverty level, and median household income determined to be approximately normally distributed. A Mann-Whitney test with significance set at *P* < .05 using the R core programming software, version 4.41, was used to compare the liver-related deaths per in-state donor use for the highest and lowest quintiles of LRM. Hawaii and Washington, DC, were omitted from comparing the quintile analysis due to their unique transplant environments secondary to vast distances from mainland transplant centers and not being a US state, respectively. However, LRM, in-state donor use as a percentage of the total number of livers received, and number of deaths per donor liver use (from in-state and all sources, respectively) for Hawaii were tabulated.^1^

Poverty rates were assessed using the US Department of Agriculture, Economic Research Service.^13^ Relative urbanization was assessed using the US Census Bureau 2020 database (Dicennial Census).^14^ Relative income was assessed using the US Census Bureau 2021 database (Median Household Income).^14,15^

## Results

### Liver-Related Mortality

**Figure 1.**
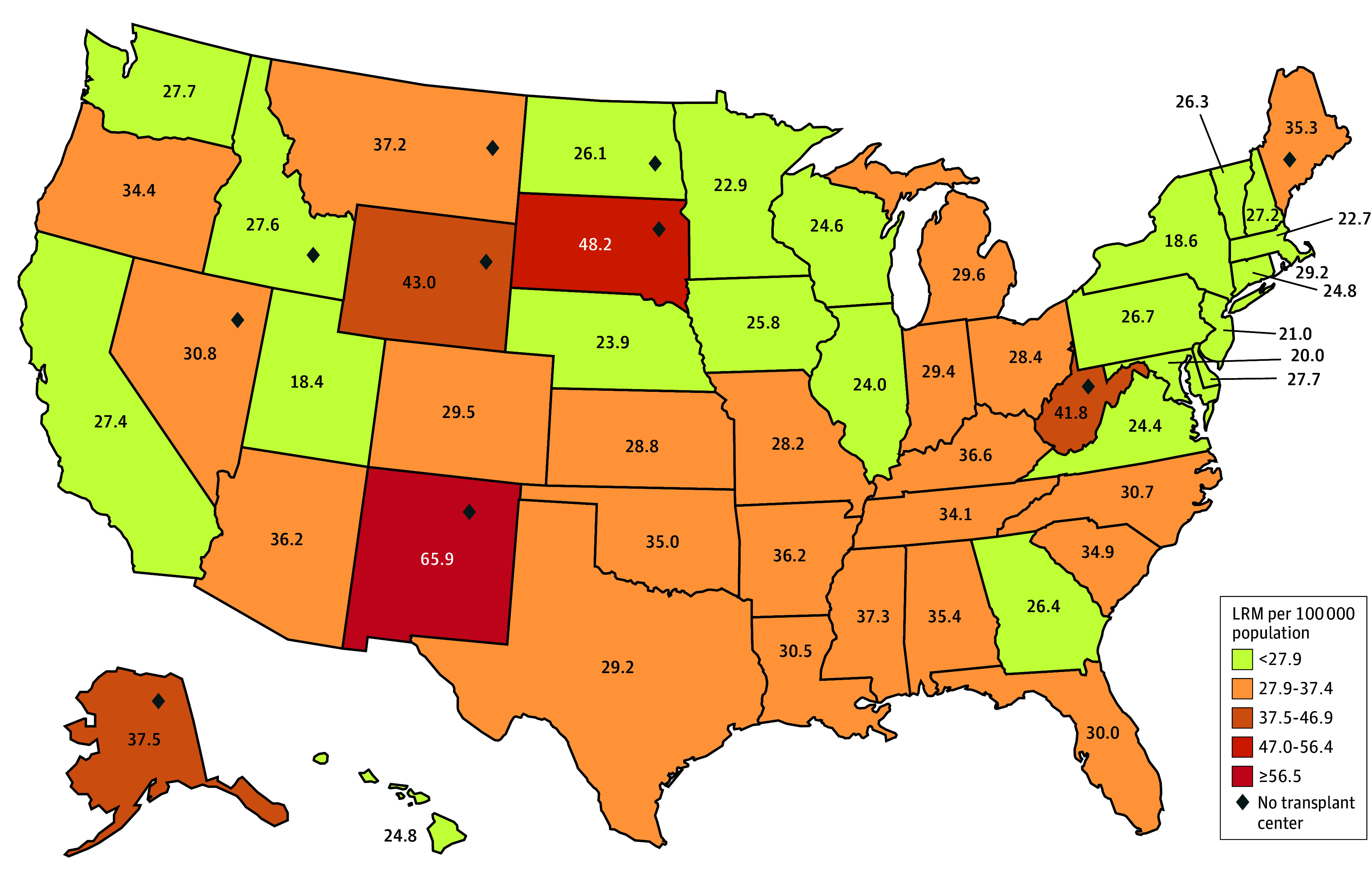
Overall Liver-Related Mortality (LRM) per State Variance of LRM in the US, organized into quintiles, is shown. Overall, LRM was 28.1 per 100 000 individuals in 2021 and increased by 19.1% during the COVID-19 pandemic. Liver-related mortality varied more than 3-fold between states, from 18.4 per 100 000 individuals per year in Utah to 65.9 per 100 000 individuals per year in New Mexico.

Overall, LRM was 93 418 (28.1/100 000 individuals) in 2021, the most recent year for which comprehensive data are available. Before the COVID-19 pandemic (2018), the overall LRM was 77 282 (23.6/100 000 individuals). Liver-related mortality thus increased by 19.1% during the COVID-19 pandemic. Liver-related mortality (Figure 1) varied more than 4-fold between states, from 18.4 per 100 000 individuals per year in Utah to 65.9 per 100 000 individuals per year in New Mexico in 2021. The correlation coefficient for LRM and waiting list rates indicated a negligible association between these metrics (*R*^2^ = 0.006).

### Liver Transplant (at Any Location) and Donor Organ Use

States with the highest LRM had a much lower rate of in-state donor transplant than states in the lowest LRM quintile (13.0% vs 35.2% in-state donors; 95% CI, 14.1%-30.3%; SE, 3.9%; *P* < .001). States with the highest proportion of liver transplants from in-state donors (57.0% for the highest quintile compared with 3.0% for the lowest quintile) had a significantly lower mean LRM (25.7 vs 32.6/100 000 people) than states with the lowest mean proportion of transplants from in-state donors (95% CI, 0.5-13.4; SE, 3.1; *P* = .03).

A marked disparity in the frequency of donor livers transplanted into residents of states other than that of the donor was also observed. Residents of states in the lowest LRM quintile received over 2 times more livers than residents from these states donated, while states in the highest LRM quintile exported 6% more livers than citizens from these states received at any location (95% CI, 0.7%-1.4%; *P* < .001). Within the highest LRM quintile, 50% of states were net liver exporters, sending 14% to 38% more livers than they received. Each state in the highest quintile of LRM received at most 26% of livers originating from in-state donors, with 70% transplanting less than 20%. In contrast, Utah (lowest LRM, 18.4/100 000 individuals) received nearly twice as many livers than its residents exported. California and Texas, the first and second most populous states, have low LRM rates but are outside of the lowest quintile. California received 8 times and Texas received 4 times as many livers as they contributed to the system.

**Figure 2.**
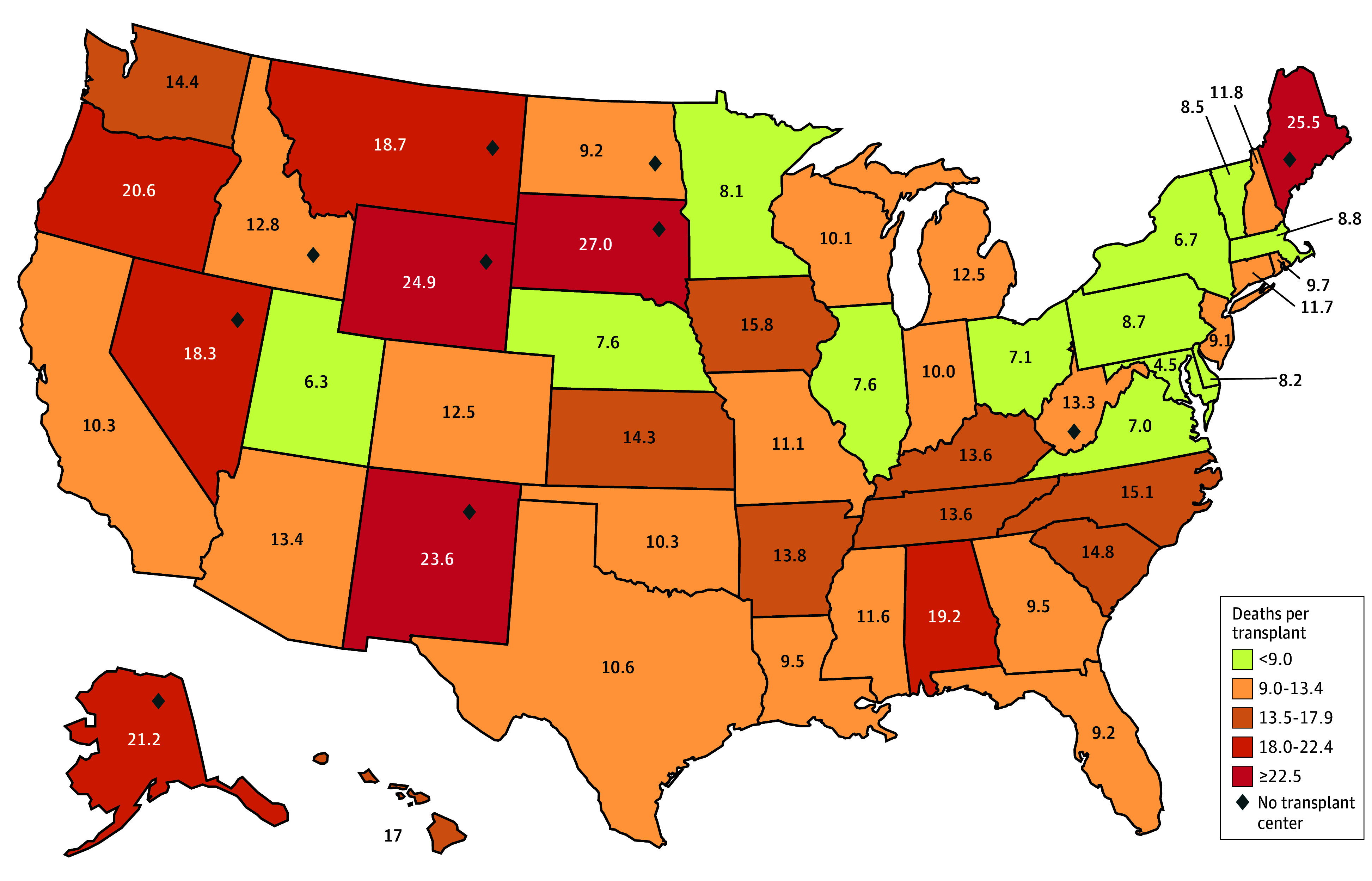
Liver-Related Deaths per All-Source Donor Use Geographic variance of liver-related deaths per liver transplanted from all sources, organized by quintiles, is shown. The mean number of liver-related deaths per transplant from all donor sources (in state and out of state) was 7.2 in the lowest LRM quintile, compared with 21.5 in the highest (95% CI, 12.1-16.6; SE, 1.1; *P* < .001).

States with higher LRM rates transplanted fewer livers donated in state, even in states with liver transplant centers. In addition, high LRM states demonstrated a significantly higher number of deaths per transplant. States with the lowest LRM rates experienced significantly fewer deaths per transplant compared with those with the highest LRM rates. The median number of deaths per transplant from livers donated in state was 26.8 (IQR, 20.5-38.6) in the lowest LRM quintile compared with a median of 109.9 (IQR, 52.1-155) in the highest quintile (95% CI, 26.1-133.2; *P* < .001). This trend held true regardless of the donor location (Figure 2). The mean number of liver-related deaths per transplant from all donor sources (in state and out of state) was 7.2 in the lowest LRM quintile compared with 21.5 in the highest (95% CI, 12.1-16.6; SE, 1.1; *P* < .001; Figure 2). In addition, residents of states within the highest quintile of liver-related deaths per transplant from in-state donors received fewer in-state donor livers for transplants, using an mean of only 5.4% of livers donated within their borders compared with 52.1% in states in the lowest quintile (95% CI, 34.3%-59.1%; SE, 5.9%; *P* < .001). Residents of states in the highest quintile of deaths per transplant from all sources transplanted a mean of 9.8% of livers donated in-state compared with 32.1% in the lowest quintile (95% CI, 10.6%-34.2%; SE, 5.6%; *P* < .001). Differences were also found between the states in the highest quintile and the states in lowest quintile of LRM in association with median household income ($61 871 vs $78 419.5; 95% CI, $7933.21-$24 833.99; SE, $3866.1; *P* < .001) and the states in the highest quintile and the states in lowest quintile of LRM in association with the percentage below the federal poverty level for all ages (57.7% vs 13.8%; 95% CI, 1.8%-6.2%; SE, 1.1%; *P* = .001), compared with 82% and 10.8%, respectively. Ten states had no liver transplant center, and 60% of states with the highest rates of LRM lack liver transplant centers.

## Discussion

The availability of critical, specialized health care should, ideally, be proportional to the relative prevalence and burden of disease. One of the most significant challenges of any health care system is to match the demand for health care services with the need for those services. This study of LRM and liver donation and use demonstrates striking geographic disparities in the US between the burden of liver disease and access to the lifesaving services of liver transplant. Rural status characterizes many of the states with higher LRM. A prior analysis of 174 630 patients who were on a waiting list and underwent a heart, liver, or kidney transplants found that individuals living in rural areas of the US were significantly less likely to be on a waiting list and receive a transplant compared with their urban counterparts.^1^ Although geospatial factors are intuitively associated with known determinants of pretransplant and posttransplant outcomes, such as cold ischemic time and geographic proximity to transplant centers, social determinants of health are also associated with marked disparities in liver transplant access. Black patients, for example, are less likely to be referred early for liver transplant evaluation and more likely to live closer to transplant centers and present at transplant with higher MELD scores.^16^ Our current findings should not be construed as a critique of organ allocation policies. Instead, the findings reported in this analysis highlight the limitations of any allocation policy to equitably reduce LRM through access to liver transplant. Instead, these observations highlight the presence and effect of disparities in access. Several aspects of the analysis bear detailed consideration, as they may shed needed insight into the scale and nature of the noted geographic disparities.

A primary finding is the several-fold variation in LRM between states. Liver transplant is a lifesaving option for approximately 10 000 patients a year in the US. Although some correlation between access to liver transplant and LRM is likely, LRM is associated with a host of factors that vary between states, including, but not limited to, dietary consumption patterns, alcohol use, and median household income, among other social determinants of health, as well as access to high-quality, local medical care. It is probable that some patients who die will have been ineligible for liver transplant (eg, due to psychosocial reasons or insurance coverage). It is certainly true that rates of alcohol-related liver deaths tend to be higher in states with higher LRM (Table 1; Table 2).^17^ Nonalcohol-related LRM is also often high in states with higher overall LRM. Data that would definitively determine the frequency of unsuitability for transplant are not readily available. Determinations of eligibility are often made by referring clinicians based on perceptions of transplant candidacy that may not reflect current listing practices of transplant centers. Transplant rates for patients with acutely decompensated alcohol use disorder–related liver disease have greatly increased, for example.^18^ Although the precise association of geographic isolation from liver transplant centers with LRM cannot be determined for a specific state, we found that 60% of states with the highest rates of LRM lack liver transplant centers. Proximity to liver transplant centers has been shown to be a major determinant of mortality among patients with chronic liver disease. In a detailed analysis of more than 16 000 patients with chronic liver disease, mortality was independently associated with distance from a liver transplant center, as a continuous variable per unit increase more than 80 km (50 miles).^2^ The variance in LRM between states demonstrated in Figure 1 suggests that investigating public health challenges at the state level is imperative, particularly for states in the highest quintiles of LRM. The most obvious impediment to liver transplant in many states with high LRM is the relative lack of liver transplant centers within their borders compared with the states in the lowest quintile of LRM. Ten states, including 5 large contiguous states, have no transplant center (Figure 1). This reality necessarily forces potential recipients to travel long distances and bear the substantial lost income and travel and related costs to access liver transplant care. For many patients with advanced liver disease, the economic barriers may be insurmountable.^19^ In an analysis of 207 liver transplant recipients surveyed on financial burden and health-related quality of life, it was found that 1 in 4 experienced a high financial burden with concomitant lower health-related quality of life.^19^ The requirement of many transplant programs for caregivers to accompany potential recipients to attend pretransplant, peritransplant, and posttransplant care exacerbates the economic barriers to transplant care.^20^ Transplant centers are frequently located in geographic areas with higher prices for lodging and food than states with high LRM and no transplant center. Funding mechanisms for supporting access to liver transplant centers would be a likely facet of any plan to improve access for patients in high LRM states.^21^

**Table 1.** State Liver-Related Mortality and Donor Use Rates

State	LRM, No. per 100 000^a^		Total No. of transplants	All-source donor use, No.^b^	Livers exported, No.^c^	Transplant centers, No.	In-state donor use/total received, %^d^
	Overall	Nonalcohol^e^					
Alabama	35.4	26.9	186	93	161	2	13.4
Alaska	37.5	16.1	23	13	21	0	8.7
Arizona	36.2	20.8	214	197	159	5	25.7
Arkansas	36.2	27.5	92	79	71	1	22.8
California	27.4	14.3	833	1046	129	14	84.3
Colorado	29.5	13.5	139	137	78	4	43.9
Connecticut	24.8	16.6	75	76	64	2	14.7
Delaware	27.7	20.9	66	34	63	1	4.6
DC	18.4	11.5	19	17	19	2	0.00
Florida	30	20.1	674	710	190	7	71.8
Georgia	26.4	19.2	280	300	174	2	37.9
Hawaii	24.8	19	16	21	4	1	75.0
Idaho	27.6	13	46	41	43	1	6.6
Illinois	24	17	345	399	206	6	40.3
Indiana	29.4	19.5	258	200	198	1	23.3
Iowa	25.8	15.1	79	52	66	1	16.5
Kansas	28.8	17.4	107	59	91	1	15.0
Kentucky	36.6	27.1	165	121	141	2	14.6
Louisiana	30.5	23.3	201	149	137	4	31.8
Maine	35.3	21.6	31	19	17	0	6.5
Maryland	20	13.9	173	271	104	2	39.9
Massachusetts	22.7	15.6	119	181	78	6	34.5
Michigan	29.6	19.6	268	238	179	4	33.2
Minnesota	22.9	11	107	162	62	2	42.1
Mississippi	37.3	23.6	108	95	81	1	25.0
Missouri	28.2	20.6	238	157	184	6	22.7
Montana	37.2	15.8	40	22	35	0	12.5
Nebraska	23.9	11.8	55	62	36	1	34.6
Nevada	30.8	16.5	123	53	119	0	3.3
New Hampshire	27.2	15.8	31	32	29	0	6.5
New Jersey	21	16.1	205	213	160	2	22.0
New Mexico	65.9	36.8	60	59	51	0	15.0
New York	18.6	13.2	402	551	222	8	44.7
North Carolina	30.7	21.4	281	214	230	3	18.2
North Dakota	26.1	11.6	15	22	11	0	26.7
Ohio	28.4	19.2	425	474	252	6	40.7
Oklahoma	35	21.3	105	135	68	2	35.2
Oregon	34.4	16.2	97	71	76	2	21.7
Pennsylvania	26.7	20.2	452	399	322	10	28.8
Rhode Island	29.2	18.4	19	33	17	0	10.5
South Carolina	34.9	23	122	123	98	1	19.7
South Dakota	48.2	16.4	12	16	13	0	0.0
Tennessee	34.1	22.3	264	175	211	3	20.1
Texas	29.2	21.3	757	815	193	13	74.4
Utah	18.4	11.5	90	97	52	3	42.2
Vermont	26.3	13.6	14	20	14	0	0.0
Virginia	24.4	17.9	213	303	144	2	32.4
Washington	27.7	12.4	174	149	86	3	50.6
West Virginia	41.8	32	87	56	82	0	5.8
Wisconsin	24.6	14.1	158	144	127	4	19.6
Wyoming	43	19	14	10	14	0	0.0
Total, mean	28.1	18.2	9077	178.7	5382	141	26.3

Abbreviation: LRM, liver-related mortality.

^a^ LRM = all-cause LRM per 100 000 population.

^b^ All-source donor use = donor organ transplanted into state recipient from in-state and out-of-state sources.

^c^ Livers exported = organs transplanted into residents of any state other than of donor residence.

^d^ In-state donor use/total received (%) = residence of donor = residence of recipient/all livers received.

^e^ Nonalcohol = nonalcohol LRM per 100 000 population.

**Table 2.** State Income, Poverty, Rural, Insurance, and COVID Vaccination Characteristics

State	Median household income, $^a^	Residents, %			
		Below the federal poverty level^b^	Rural	Insured by Medicaid^b^	At least 1 dose of COVID-19 vaccination^c^
Alabama	54 943	16.1	42.1	19.4	58.5
Alaska	80 287	10.5	40.0	24.2	65.0
Arizona	65 913	12.8	11.1	21.4	67.3
Arkansas	52 123	16.3	45.3	27.2	62.7
California	84 097	12.3	6.6	26.6	80.0^d^
Colorado	80 184	9.7	15.6	18.7	74.6
Connecticut	83 572	10.1	13.5	22.5	88.6
Delaware	72 724	11.6	15.6	20.9	76.7
DC	93 547	16.5	0.0	24.9	88.5
Florida	61 777	13.1	8.0	17.9	74.5
Georgia	65 030	14	26.6	18.0	61.3
Hawaii	88 005	11.2	15.3	20.8	83.7^d^
Idaho	63 377	11	33.1	20.2	52.1
Illinois	72 563	12.1	13.7	19.7	72.0
Indiana	61 944	12.2	28.0	20.1	57.9
Iowa	65 429	11.1	36.9	20.4	64.9
Kansas	64 521	11.7	28.6	14.9	69.3
Kentucky	55 454	16.5	41.4	28.7	62.5
Louisiana	53 571	19.6	28.3	32.0	57.4
Maine	63 182	11.5	63.6	19.9	85.9
Maryland	91 431	10.3	14.3	20.1	80.5
Massachusetts	89 026	10.4	8.7	23.1	90.7
Michigan	63 202	13.1	28.4	23.5	63.5
Minnesota	77 706	9.3	31.1	18.1	71.4
Mississippi	49 111	19.4	53.5	24.1	55.3
Missouri	61 043	12.7	30.6	15.1	62.3
Montana	60 560	11.9	48.1	20.2	62.1
Nebraska	66 644	10.8	29.0	14.7	66.4
Nevada	65 686	14.1	6.3	20.5	69.5
New Hampshire	83 449	7.2	43.7	13.5	84.0^d^
New Jersey	89 703	10.2	6.0	18.4	83.7
New Mexico	54 020	18.4	26.0	33.6	80.7
New York	75 157	13.9	14.5	27.7	84.0
North Carolina	60 516	13.4	34.3	18.7	76.4
North Dakota	68 131	11.1	39.4	12.0	62.2
Ohio	61 938	13.4	22.2	21.5	60.5
Oklahoma	56 956	15.6	35.3	20.2	66.0
Oregon	70 084	12.2	20.5	23.5	74.0
Pennsylvania	67 587	12.1	24.1	20.8	73.4
Rhode Island	74 489	11.4	9.1	23.9	78.2
South Carolina	58 234	14.6	31.8	20.0	89.0
South Dakota	63 920	12.3	43.2	13.7	62.8
Tennessee	58 516	13.6	34.2	19.9	70.9
Texas	67 321	14.2	17.0	17.0	58.7
Utah	79 133	8.6	12.3	11.3	66.8
Vermont	67 674	10.3	68.8	23.1	67.4
Virginia	80 615	10.2	26.6	15.5	89.3
Washington	82 400	9.9	17.5	21.2	76.0
West Virginia	50 884	16.8	54.6	28.2	75.7
Wisconsin	67 080	10.8	35.5	18.2	61.9
Wyoming	68 002	11.4	39.5	11.8	68.2

^a^ Median household income = estimated median household income (2021) in the past 12 months (in 2021 inflation-adjusted dollars).

^b^ Source: US Census Bureau, 2021 American Community Survey 1-Year Estimates.^17^

^c^ Percentage of residents with at least 1 dose of COVID-19 vaccine on December 30, 2021.

^d^ Closest date for which data are available: California (December 8, 2021), Hawaii (January 3, 2022), and New Hampshire (November 20, 2021); December 8, 2021 is closest available data point.

Another factor potentially associated with the observed disparities in LRM, liver transplant access, and donor organ use rates could be population size and wealth variation between states. The states in the highest quintile of LRM tend to have significantly lower population sizes and lower real median household incomes than those in the lowest quintile of LRM. The skillsets required to establish and maintain high-performing liver transplant centers are scarce and resource intensive. Attracting the needed personnel to rural areas, which are likely to be relatively low in volume and compensation, is clearly challenging. Whether organ allocation policies should account for the risk associated with geographic isolation from transplant centers merits exploring.

Finally, because these data were accrued during the COVID-19 pandemic, an association of COVID-19 with overall LRM and nonalcohol-related mortality is possible. The correlation coefficient of overall LRM with respect to COVID-19 vaccination rate was low for overall LRM and nonalcohol-related LRM (*R*^2^ = 0.08 and 0.01, respectively), indirectly suggesting a weak association, if any, between COVID-19 disease and LRM variation between states.

### Limitations

Our study has several limitations. One is the well-described variance in perceived and actual cause of death as recorded on death certificates, the source documents for the causes of death in the CDC WONDER database.^22^ The information and confounding biases may have also been present to unknown degrees. The scale and breadth of these databases are likely to reduce the effect of these potential biases.

## Conclusions

This cohort study found that LRM rates have increased dramatically since the COVID-19 pandemic and vary several-fold between states. Liver transplant rates are paradoxically lowest among residents living in states with the highest LRM. Although these observations do not establish and are not intended to imply cause and effect, the observations are nonetheless interesting and, at minimum, likely reflect the relative lack of access to liver transplants in states with high LRM. These findings highlight apparent geographic disparities in access to liver transplants that allocation policy cannot address, raising important questions about the need for new strategies to ensure fair and balanced access to liver transplants for all patients, regardless of their location.

## Notes

Supplement.
Data Sharing Statement
