# Supplementary material for: Current Burden of and Geographic Disparities in Liver Mortality and Access to Liver Transplant
Source: JAMA Netw Open. 2024 Oct 18;7(10):e2439846. doi: 10.1001/jamanetworkopen.2024.39846 (PMC11581511; doi:10.1001/jamanetworkopen.2024.39846)
Supplement: Supplement. — Data Sharing Statement [file jamanetwopen-e2439846-s001.pdf]

# Data Sharing Statement

Rinella. Current Burden of and Geographic Disparities in Liver Mortality and Access to Liver Transplant. *JAMA Netw Open*. Published October 18, 2024.
doi:10.1001/jamanetworkopen.2024.39846

**Data**
**Data available:** Yes
**Data types:** Deidentified participant data
**How to access data:** mcharlton@medicine.bsd.uchicago.edu
**When available:** With publication

**Supporting Documents**
**Document types:** None

**Additional Information**
**Who can access the data:** anyone requesting the data
**Types of analyses:** for any purpose
**Mechanisms of data availability:** with investigator support
